# Comparison of catheter-related bloodstream infection between peripherally inserted central catheters and tunneled central venous catheters in patients receiving home parenteral nutrition: a meta-analysis

**DOI:** 10.3389/fnut.2026.1742418

**Published:** 2026-02-17

**Authors:** Yu-Li Zheng, Ying Wang, Shu-Ping Qi, Wei Zhang, Pei-Yan Lin

**Affiliations:** 1 Department of Oncology, Zhejiang Hospital, Hangzhou, China; 2 Department of Gynecology, Zhejiang Hospital, Hangzhou, China; 3 Department of General Practice, Zhejiang Hospital, Hangzhou, China

**Keywords:** central venous catheters, CRBSI, homeparenteral nutrition, meta-analysis, peripherally inserted central catheters

## Abstract

**Background:**

Catheter-related bloodstream infection (CRBSI) remains one of the most severe complications in patients receiving home parenteral nutrition. Tunneled central venous catheters (CVCs) and peripherally inserted central catheters (PICCs) are the most commonly used devices for home parenteral nutrition. However, the relative risks of CRBSI from these devices remain controversial. This meta-analysis aimed to compare the incidence of CRBSI between PICCs and tunneled CVCs in patients receiving home parenteral nutrition.

**Methods:**

A systematic search of PubMed, Embase, and the Cochrane Library databases was conducted from database inception to 3 June 2025 to identify studies comparing the incidence of CRBSI between PICCs and tunneled CVCs in patients receiving home parenteral nutrition. Pooled risk ratios (RRs) with 95% confidence intervals (CIs) were calculated to assess the relative risk of CRBSI associated with PICCs versus tunneled CVCs using either the fixed-effects model or the random-effects model. The certainty of evidence was assessed using the GRADE approach.

**Results:**

A total of 10 articles, involving 1,139 patients with PICCs or tunneled CVCs, were included in the meta-analysis. The mean CRBSI rate was 0.77 per 1,000 PICC-days and 1.01 per 1,000 tunneled CVC-days. The pooled analysis demonstrated that PICCs were associated with a significantly lower risk of CRBSI compared with tunneled CVCs (RR:0.40, 95%CI:0.33–0.49). Subgroup analyses stratified by study design, patient population, and CRBSI definition yielded consistent results, confirming the robustness of the primary findings. According to the GRADE approach, the quality of evidence was very low for the CRBSI rate.

**Conclusion:**

PICCs were associated with a lower risk of CRBSI than tunneled CVCs in patients receiving home parenteral nutrition. However, the certainty of evidence was very low; therefore, these findings should be interpreted with caution, and further high-quality studies are needed.

## Introduction

Home parenteral nutrition is a life-sustaining therapy for patients with chronic intestinal failure or severe diseases who are unable to maintain adequate nutritional intake via oral or enteral routes (1, 2). Central venous access is indispensable for home parenteral nutrition, as it enables the long-term administration of nutrient solutions (3, 4). However, catheter-related bloodstream infection (CRBSI) remains the most frequent and serious complication associated with central venous access (5). CRBSI not only increases morbidity and hospital readmission rates but also imposes a substantial economic burden on patients and healthcare systems (6, 7).

In recent years, peripherally inserted central catheters (PICCs) have been increasingly adopted because of their relative ease of insertion, cost-effectiveness, and suitability for long-term use (8). Nevertheless, the risk of CRBSI associated with PICCs compared with conventional central venous catheters (CVCs) remains controversial (9, 10). Some observational studies have suggested that PICCs may be associated with similar or even higher infection rates than tunneled CVCs, while other studies have reported a lower risk of CRBSI with PICCs than with tunneled CVCs (11–14). These inconsistencies highlight the need for a systematic evaluation to clarify the relative risk between PICCs and tunneled CVCs in patients receiving home parenteral nutrition. Therefore, this study aimed to conduct a comprehensive meta-analysis to compare the incidence of CRBSI between PICCs and tunneled CVCs in patients receiving home parenteral nutrition. By synthesizing the available evidence, we aimed to generate more reliable data to inform clinical practice.

## Methods

### Literature search strategy

The Preferred Reporting Items for Systematic Reviews and Meta-Analyses (PRISMA) guideline was used to conduct this systematic review and meta-analysis (15). The study protocol was registered on INPLASY (Registration number: INPLASY2025120072). A systematic literature search was conducted in PubMed, Embase, and the Cochrane Library databases from their inception to 3 June 2025. The search terms included combinations of the following keywords and Medical Subject Headings (MeSH): “peripherally inserted central catheter,” “PICCs,” “central venous catheter,” “CVCs,” and “home parenteral nutrition.” The detailed search strategy was described in Supplementary Table S1. The search was restricted to studies published in English. Reference lists of relevant articles and reviews were also manually screened to identify additional eligible studies.

### Inclusion and exclusion criteria

Studies that met the following criteria according to the PICOS guidelines were included: Patients: adult patients (≥18 years) receiving home parenteral nutrition; Intervention and comparison: peripherally inserted central catheters versus tunneled central venous catheters; Outcomes: the incidence of CRBSI; and Study design: cohort or case–control studies. Articles were excluded if they lacked eligible data or were case reports, reviews, or conference abstracts.

### Data extraction and quality assessment

All eligible data were independently extracted by two investigators using a standardized data extraction form. Any discrepancies were resolved through discussion, and if consensus could not be reached, a third investigator was consulted. Data extracted included the first author, year of publication, country, study design, treatment regimen, outcome measures, and definition of CRBSI. The methodological quality of the included studies was independently assessed by two reviewers using the Newcastle–Ottawa Scale (NOS) (16). Studies with an accumulated score of ≥6 points were considered high quality. The certainty of evidence and strength of recommendations were evaluated using the GRADE approach (17), which rates confidence in summary effect estimates across four levels: very low, low, moderate, and high.

### Statistical analysis

Stata 12.0 (STATA Corp, College Station, TX, USA) was used for the meta-analysis. Pooled risk ratios (RRs) with 95% confidence intervals (CIs) were calculated to estimate the incidence of CRBSI using either the fixed-effects model or random-effects model, depending on the degree of heterogeneity. Heterogeneity was assessed using Cochran’s *Q* test and quantified with the *I*^2^ statistic, with *I*^2^ > 50% considered substantial. Sensitivity analysis was conducted by sequentially removing individual studies to assess the robustness of the results. Subgroup analyses were conducted based on available variables. Publication bias was evaluated using funnel plots, Egger’s regression test, and Begg’s test.

## Results

### Literature selection

**Figure 1:**
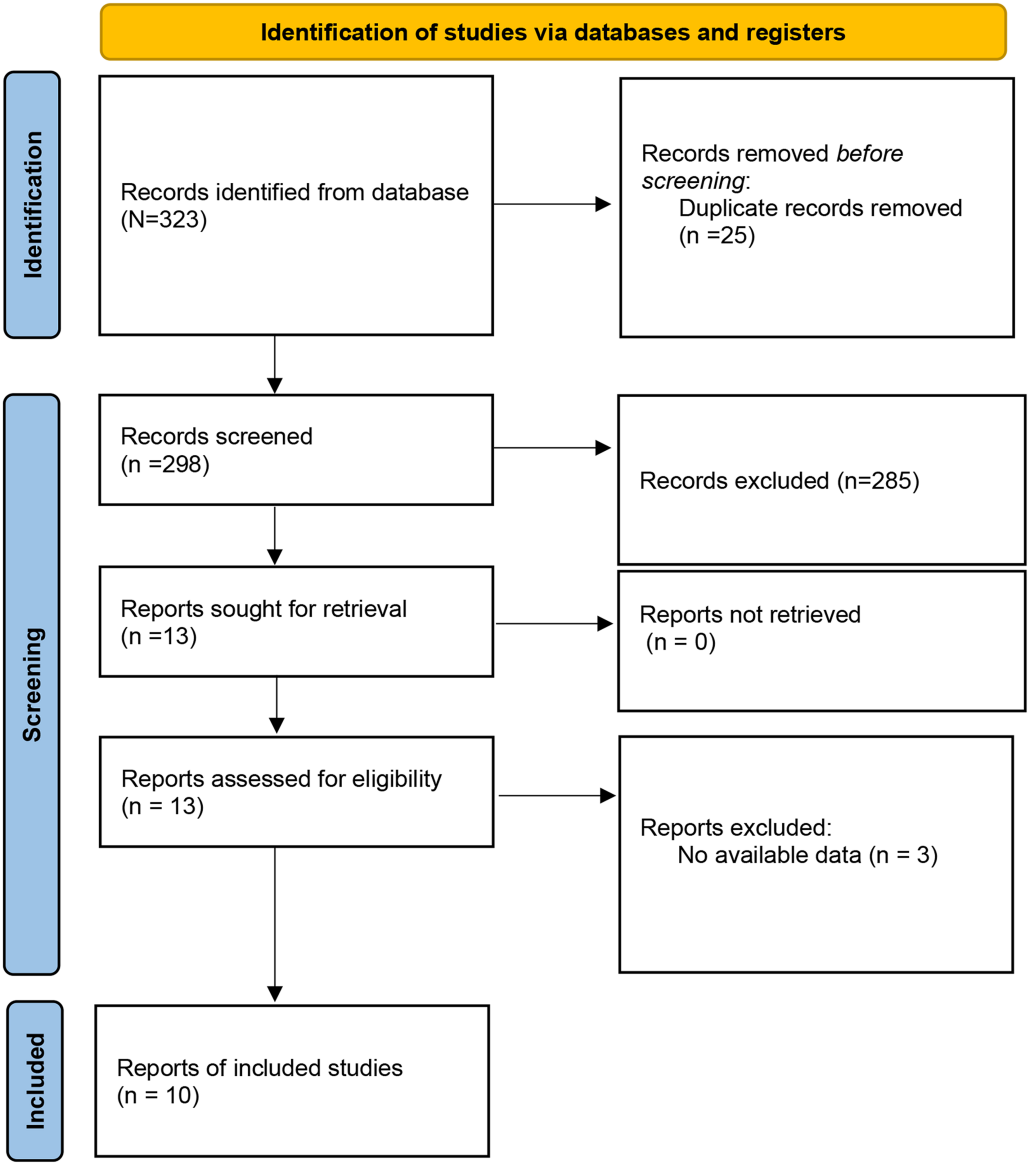
Flow diagram of literature search.

A total of 323 studies were initially retrieved from the databases. After removal of duplicates and screening titles and abstracts, 13 articles were retained for full-text review. Of these, three studies were excluded due to unavailable or insufficient data. Finally, 10 studies (11–15, 18–22) met the inclusion criteria for the meta-analysis. The study selection process is presented in the PRISMA flow diagram (Figure 1). The included 10 studies, published between 2013 and 2021, involved a total of 1,139 patients receiving PICCs, with sample sizes ranging from 48 to 202 participants. The studies were conducted between 2013 and 2021 across multiple countries, including the USA, Spain, Canada, France, Italy, Denmark, and Poland. Both prospective and retrospective cohort designs were represented, with patient populations including both cancer and non-cancer patients. CRBSI definitions varied among studies: the Infectious Diseases Society of America (IDSA) criteria were used in two studies, the CDC criteria in five studies, and no standard criteria were reported in three studies. CRBSI was generally diagnosed based on blood cultures or catheter lumen cultures, with slight differences depending on the study protocol. Catheter types included PICCs and tunneled CVCs. The number of lumens, catheter-days, and CRBSI rates per 1,000 catheter-days were reported when available (Table 1). The microorganisms responsible for catheter-associated infections are primarily Gram-positive bacteria, Gram-negative pathogens, and fungi (Table 2). The quality of the included literature was scored 6–7 (Supplementary Table 2).

**Table 1:** Characteristics of included studies.

First author	Year	Country	Study design	Mean age	Patient population	CRBSI definition	Method for CRBSI diagnosis	Catheter type	Catheter lumens	Lock solutions	Catheter-days		CRBSI per catheter-days		CRBSI events			
											PICC	CVC	PICC	CVC	PICC		CVC	
															Yes	No	Yes	No
Vashi	2017	USA	Retrospective cohort study	53.7	Cancer	IDSA	Blood cultures	PICC, Tunneled	NR	2% chlorhexidine	–	–	–	–	7	184	1	10
Santacruz	2018	Spain	Prospective cohort study	58	Cancer and non-cancer	CDC	Catheter lumen and the blood peripherally cultures	PICC, tunneled	SL:110 ML:23	NR	20,495	4,176	0.15	0.72	3	113	3	15
Botella-carretero	2013	Spain	Prospective cohort study	58.46	Cancer and non-cancer	CDC	Catheter lumen and the blood peripherally cultures	PICC, Hickman	SL:42 ML:16	NR	1,291	985	0	1.02	0	48	1	9
Elfassy	2015	Canada	Retrospective cohort study	49.2	Cancer and non-cancer	No standard criteria	Blood cultures	PICC, Hickman	NR	NR	18,907	18,191	1.96	1.93	37	165	35	27
Touré	2014	France	Prospective cohort study	55.6	Cancer and non-cancer	CDC	Blood cultures	PICC, Tunneled	NR	NR	12,322	36,812	1.38	1.82	17	66	67	54
Durkin	2016	USA	Prospective cohort study	54	Cancer and non-cancer	CDC	Blood cultures	PICC, Hickman	NR	NR	–	–	–	–	20	56	13	7
Cotogni	2015	Italy	Prospective cohort study	67	Cancer	IDSA	Catheter lumen and the blood peripherally cultures	PICC, Tunneled	All SL	2% chlorhexidine	11,504	7,835	0	0.65	0	65	5	45
Christensen	2015	Denmark	Retrospective cohort study	64.5	Cancer and non-cancer	No standard criteria	Blood cultures	PICC, Hickman	NR	NR	15,974	54,912	1.63	0.56	26	100	49	120
Xue	2020	USA	Prospective cohort study	54	Cancer and non-cancer	CDC	Catheter lumen and the blood peripherally cultures	PICC, Tunneled	SL:18 ML:95	NR	–	–	–	–	14	68	16	15
Konrad	2021	Poland	Prospective cohort study	63.5	Cancer and non-cancer	No standard criteria	Blood cultures	PICC, Tunneled	All SL	2% chlorhexidine	23,045	43,789	0.3	0.41	7	143	18	105

CRBSI, catheter-related bloodstream infection; PICC, peripherally inserted central catheter; CVC, central venous catheter; CDC, Centers for Disease Control; IDSA, Infectious Diseases Society of America; NR, not applicable.

**Table 2:** Microorganism.

First author	Year	Microbiological confirmation
Vashi	2017	*Staphylococcus epidermidis*; coagulase-negative *Staphylococci*; methicillin-resistant *Staphylococcus aureus* (MRSA); *Klebsiella pneumoniae*; *Escherichia coli*; *Enterococcus faecium*
Santacruz	2018	*Klebsiella pneumoniae*; *Staphylococcus epidermidis*; coagulase-negative *Staphylococci*
Botella-Carretero	2013	Coagulase-negative *Staphylococci*; *Staphylococcus* spp.; *Actinomyces* spp.; *Enterococcus faecalis*
Elfassy	2015	*Candida parapsilosis*; *Candida glabrata*; *Candida albicans*; coagulase-negative *Staphylococci*; *Staphylococcus* *aureus*; *Enterococcus faecalis*; diphtheroid bacilli
Touré	2014	Coagulase-negative *Staphylococci*; *Staphylococcus aureus*; *Streptococcus* spp.; *Bacillus* spp.; *Enterococcus* *faecalis*; *Enterobacter* spp.; *Escherichia coli*; *Klebsiella* spp.; *Acinetobacter* spp.; *Pseudomonas* spp.; *Serratia* *marcescens*; *Stenotrophomonas maltophilia*; *Candida albicans*
Durkin	2016	*Staphylococcus epidermidis*; *Candida* spp.; *Klebsiella* spp.; *Enterococcus faecalis*; methicillin-sensitive *Staphylococcus aureus* (MSSA); methicillin-resistant *Staphylococcus aureus* (MRSA); other coagulase-negative *Staphylococci*
Cotogni	2015	*Staphylococcus aureus*; coagulase-negative *Staphylococci*; *Enterococcus* spp.; *Escherichia coli*; *Enterobacter* *cloacae*; *Klebsiella pneumoniae*
Christensen	2015	*Staphylococcus aureus*; coagulase-negative *Staphylococci*; *Enterococcus faecalis*; *Bacillus cereus*; *Corynebacterium* spp.; *Streptococcus salivarius*; *Micrococcus luteus*; *Enterobacter* spp.; *Escherichia coli*; *Acinetobacter* spp.; *Pseudomonas aeruginosa*; *Stenotrophomonas maltophilia*; *Klebsiella* spp.; *Candida albicans*
Xue	2020	NR
Konrad	2021	NR

NR, not reported.

### Meta-analysis of CRBSI

**Figure 2:**
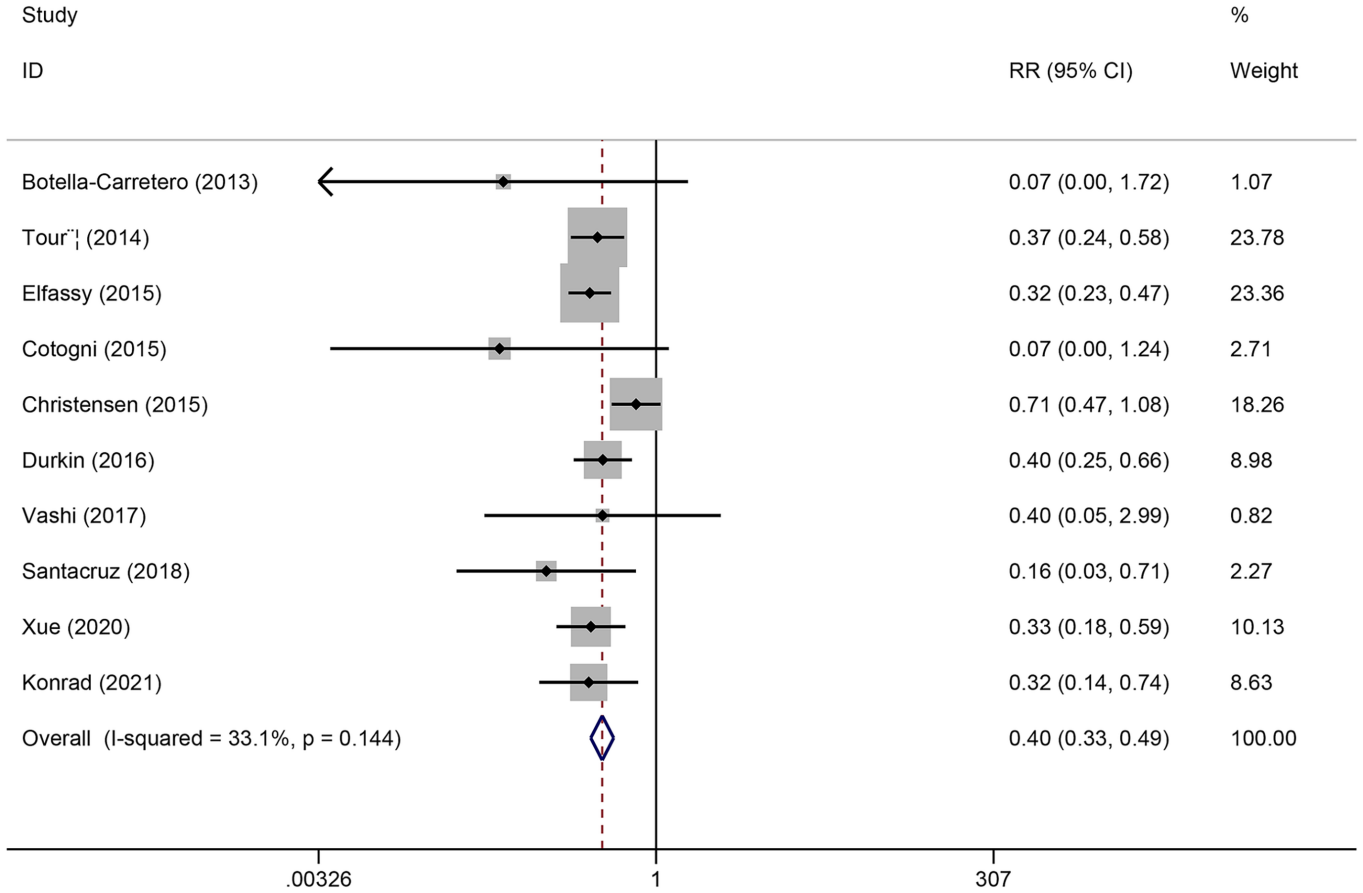
Forest plot for the incidence of CRBSI between PICCs and tunneled CVCs groups.

Ten studies reported the effects of PICCs and tunneled CVCs on the incidence of CRBSI based on per-patient risk. Moderate heterogeneity was observed among the included studies (*I*^2^ = 33.1%, *p* = 0.14). Therefore, both fixed-effects and random-effects models were applied. Under both models, PICC use was associated with a significantly lower risk of CRBSI compared with tunneled CVCs (fixed-effects model: RR = 0.40, 95% CI 0.33–0.49; random-effects model: RR = 0.38, 95% CI 0.29–0.50; both *p* < 0.001) (Figure 2). According to GRADE, the quality of evidence was very low (Supplementary Table 3). In addition, seven studies reported CRBSI incidence based on per 1,000 catheter-days. The mean CRBSI rate was 0.77 per 1,000 PICC-days (range: 0.00–1.96 per 1,000 PICC-days) and 1.01 per 1,000 tunneled CVC-days (range: 0.41–1.93 per 1,000 tunneled CVC-days), indicating a consistently lower incidence associated with PICCs.

Subgroup analysis by country showed consistent results across most regions, including the USA, Spain, Canada, France, and Denmark, with no significant differences between PICCs and tunneled CVCs. In contrast, Italy and Poland did not demonstrate a statistically significant difference between the two catheter types. Subgroup analyses stratified by study design demonstrated that both prospective cohort and retrospective cohort studies found a significant difference between PICCs and tunneled CVCs, with PICC use being associated with a lower risk of CRBSI. When stratified by patient population, the association between catheter type and CRBSI risk remained consistent in both cancer and non-cancer populations. Moreover, subgroup analyses stratified by CRBSI definition demonstrated that studies using IDSA criteria, CDC criteria, and those applying non-standard clinical definitions all showed a statistically significant difference between PICCs and tunneled CVCs, with PICC use consistently associated with a lower risk of CRBSI (Table 3).

**Table 3:** Subgroup analysis.

Subgroup	Group	No. studies	Fixed-effects model		Random-effects model	
			RR (95% CI)	*p*-value	RR (95% CI)	*p*-value
Country	USA	3	0.37 (0.25, 0.54)	0.001	0.37 (0.26, 0.54)	0.001
	Spain	2	0.13 (0.03, 0.51)	0.003	0.14 (0.03, 0.53)	0.004
	Canada	1	0.32 (0.23, 0.47)	0.001	0.32 (0.23, 0.47)	0.001
	France	1	0.37 (0.24, 0.58)	0.001	0.37 (0.24, 0.58)	0.001
	Italy	1	0.07 (0.00, 1.24)	0.070	0.07 (0.00, 1.24)	0.070
	Denmark	1	0.71 (0.47, 1.08)	0.109	0.71 (0.47, 1.08)	0.109
	Poland	1	0.32 (0.14, 0.74)	0.008	0.32 (0.14, 0.74)	0.008
Design	Prospective cohort	7	0.33 (0.25, 0.44)	0.001	0.47 (0.24, 0.93)	0.030
	Retrospective cohort	3	0.49 (0.38, 0.65)	0.001	0.35 (0.27, 0.45)	0.001
Population	Cancer	2	0.15 (0.03, 0.81)	0.028	0.21 (0.03, 1.37)	0.105
	Non-cancer	8	0.41 (0.34, 0.50)	0.001	0.39 (0.30, 0.51)	0.001
CRBSI definition	IDSA	2	0.15 (0.03, 0.81)	0.028	0.22 (0.03, 1.38)	0.105
	CDC	5	0.35 (0.26, 0.47)	0.001	0.36 (0.27, 0.47)	0.001
	No standard criteria	3	0.46 (0.36, 0.60)	0.001	0.43 (0.24, 0.78)	0.001

CRBSI, catheter-related bloodstream infection; CDC, Centers for Disease Control; IDSA, Infectious Diseases Society of America.

### Sensitivity analysis

**Figure 3:**
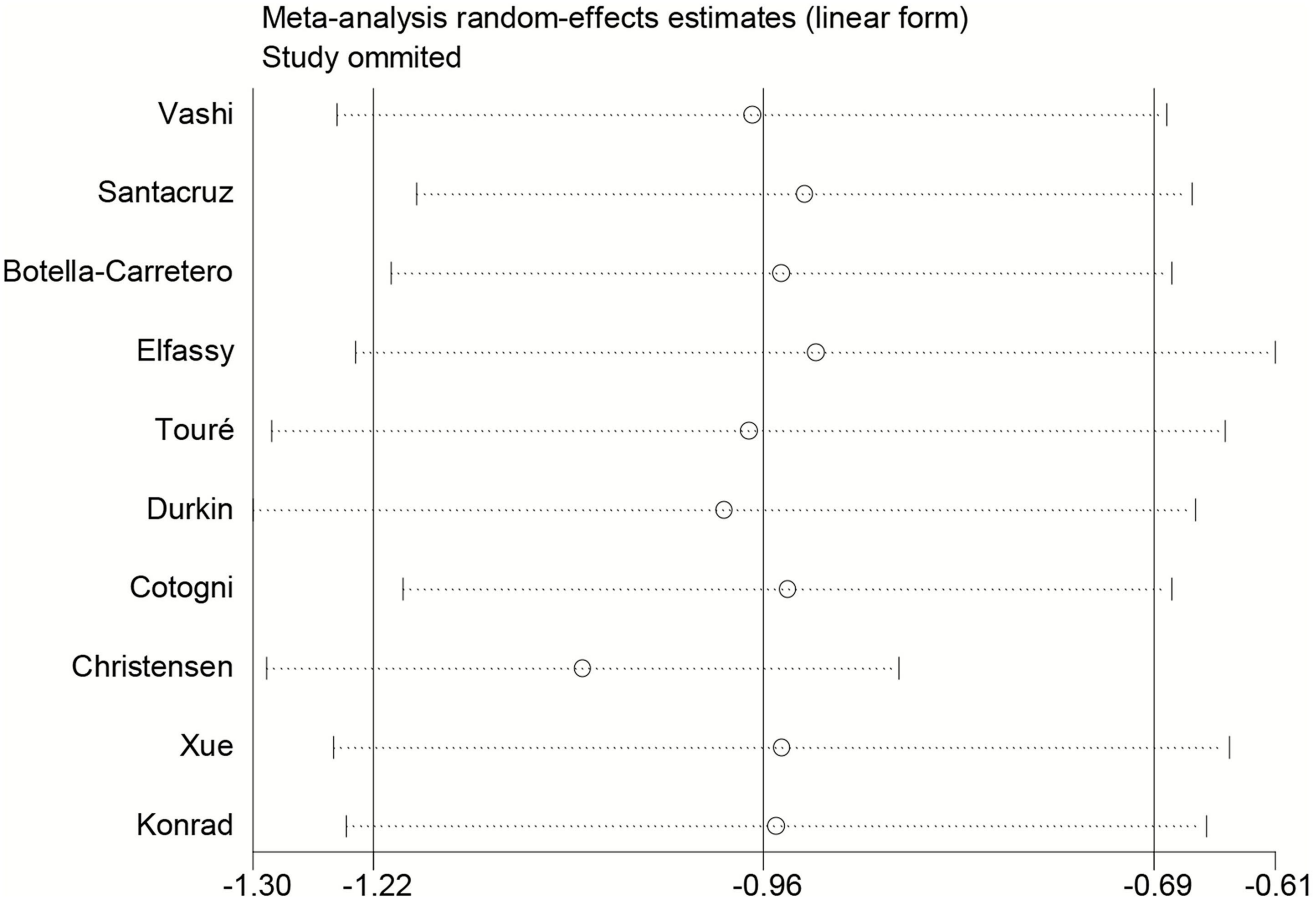
Sensitivity analysis for CRBSI risk.

**Figure 4:**
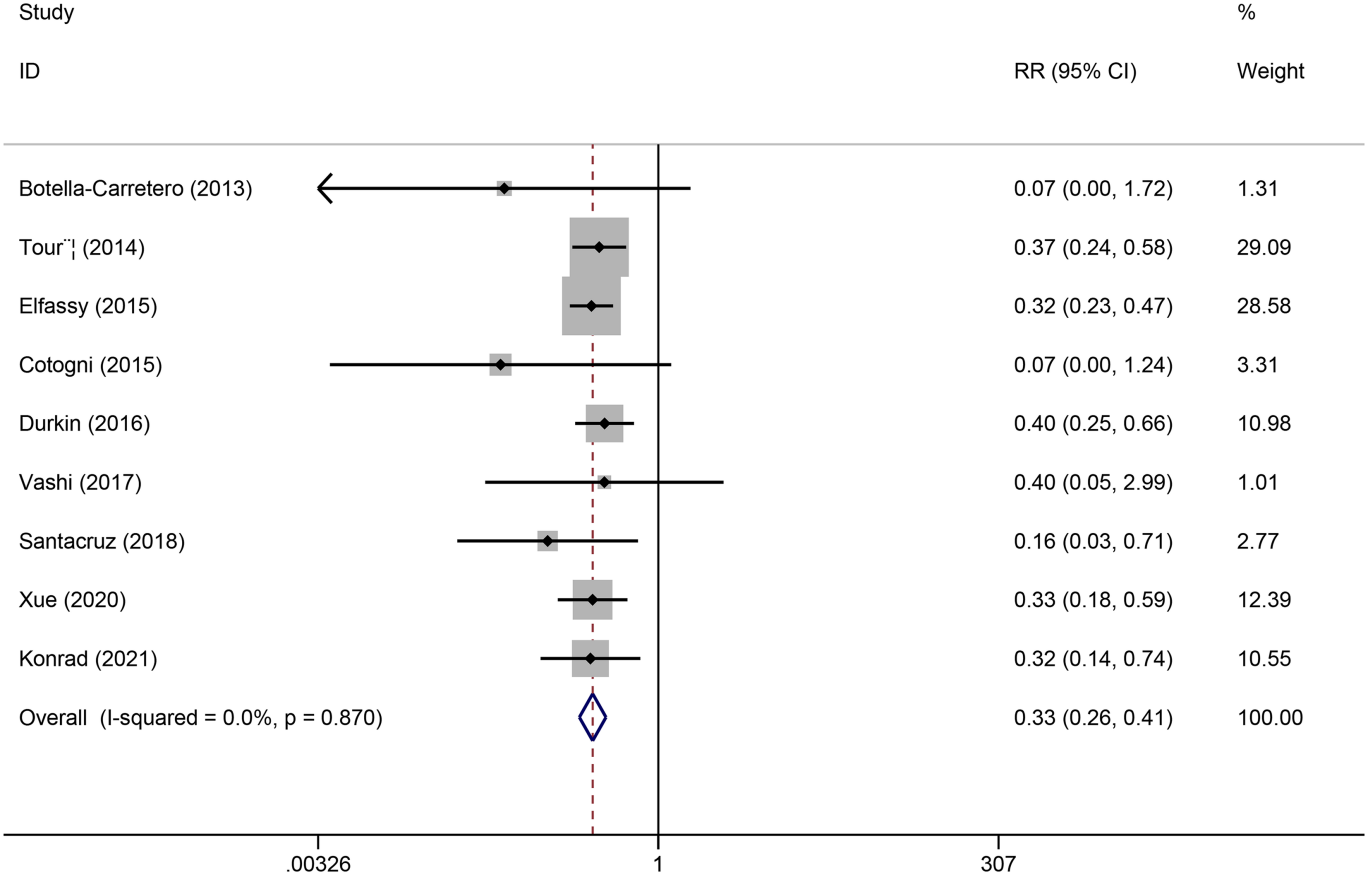
Forest plot of the incidence of CRBSI comparing PICCs and tunneled CVCs after removal of one study.

Sensitivity analysis was performed by sequentially excluding each study (Figure 3). After excluding the study by Christensen et al., heterogeneity was eliminated (*I*^2^ decreased from 33.1 to 0%). The pooled effect estimate remained stable, with PICCs still associated with a significantly lower risk of CRBSI compared with tunneled CVCs (RR = 0.33, 95% CI 0.26–0.41), indicating the robustness of the overall findings (Figure 4).

### Publication bias

**Figure 5:**
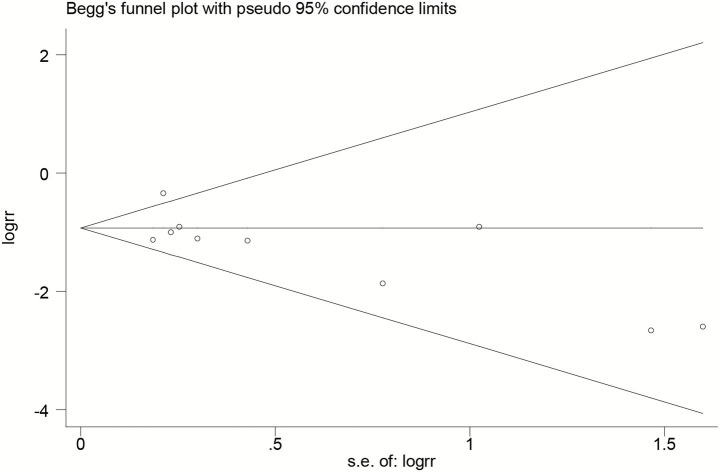
Begg’s funnel plot for publication bias.

Publication bias was evaluated using funnel plots and Begg’s test. Visual inspection of the funnel plot revealed no significant asymmetry (Figure 5). Both Egger’s test (*p* = 0.138) and Begg’s test (*p* = 0.421) indicated no significant publication bias.

## Discussion

The choice between PICCs and tunneled CVCs for home parenteral nutrition remains a subject of an important clinical debate. This controversy arises from differences in complication risks, patient comfort, and cost-effectiveness. Therefore, determining which catheter insertion method provides superior outcomes is of critical importance. In this meta-analysis, we found that PICCs may be associated with a lower risk of CRBSI compared with tunneled CVCs in patients receiving home parenteral nutrition.

The observed lower risk of CRBSI associated with PICCs may be explained by several plausible reasons. First, peripheral insertion may reduce the risk of procedural contamination because the puncture site is more accessible and easier to maintain aseptically (23, 24). Second, PICC placement is generally less invasive and often performed at the bedside using ultrasound guidance, which has been associated with fewer insertion-related complications and shorter procedure times—factors that may reduce infection risk (25, 26). In addition, catheter design characteristics may contribute to differences in CRBSI risk. PICCs used for home parenteral nutrition are more frequently single lumen and of smaller diameter, features that have been associated with lower rates of intraluminal colonization (27, 28). However, tunneled CVCs are more frequently selected for patients with greater disease severity, poor peripheral venous access, or anticipated prolonged or complex therapy (29, 30). As a majority of the included studies were observational and lacked adjustment for illness severity, catheter indication, and functional status, residual confounding cannot be excluded.

Our findings are largely consistent with previous observational studies reporting a lower incidence of CRBSI with PICCs than with tunneled CVCs, particularly in home-based care or long-term use scenarios (11, 13, 21). Multiple prospective cohort studies have shown that PICCs are associated with fewer infectious complications, shorter catheter dwell-related morbidity, and lower hospitalization rates (14, 18, 19, 22). In contrast, studies conducted in hospital settings sometimes reported minimal differences, suggesting that contextual factors, such as infection prevention training, institutional protocols, and healthcare personnel expertise, may influence the observed outcomes (31). These comparisons underscore that, while catheter selection is important, strict adherence to infection control measures remains essential. Notably, the diagnosis of CRBSI varied across the included studies. CRBSI is defined according to established criteria, such as those proposed by the Centers for Disease Control and Prevention (CDC) (32) or the Infectious Diseases Society of America (IDSA) (33). However, several studies relied on less stringent definitions, such as positive blood cultures without standardized confirmation methods (18, 19, 21). This inconsistency in diagnostic criteria may have contributed to heterogeneity in the pooled estimates and should be considered when interpreting the results.

Despite the important insights provided by our study, several limitations should be considered. First, a majority of the included studies were observational, introducing the potential for selection bias and residual confounding. Second, substantial heterogeneity existed across studies in CRBSI definitions, diagnostic criteria, and surveillance methods, which may have influenced the pooled estimates. Third, the majority of studies lacked detailed reporting of catheter-related variables, including catheter dwell time, the number of lumens, comorbidities, or concurrent medications, all of which can affect infection risk. Finally, the lack of high-quality randomized controlled trials limits the generalizability of our conclusions, particularly across diverse healthcare settings and populations with different home care practices. Importantly, when assessed using the GRADE framework, the overall certainty of evidence for the CRBSI was very low, due to the risk of bias, inconsistency, and imprecision.

Although the results of this meta-analysis indicate that PICCs may be associated with a lower risk of CRBSI compared with tunneled CVCs in patients receiving home parenteral nutrition, the available studies are observational, carrying a substantial risk of bias and residual confounding, which led to a very low certainty of evidence. High-quality, well-designed randomized controlled trials with standardized CRBSI definitions and comprehensive reporting of catheter-related variables are urgently needed to confirm these findings and to inform optimal vascular access strategies in home parenteral nutrition care.

## Data Availability

The original contributions presented in the study are included in the article/Supplementary material; further inquiries can be directed to the corresponding author.
