# Supplementary material for: Comparison of catheter-related bloodstream infection between peripherally inserted central catheters and tunneled central venous catheters in patients receiving home parenteral nutrition: a meta-analysis
Source: Front Nutr. 2026 Feb 17;13:1742418. doi: 10.3389/fnut.2026.1742418 (PMC12953089; doi:10.3389/fnut.2026.1742418)
Supplement: Supplementary file 1 [file Table_1.DOCX]

**Table S1：The details of the search strategies.**

|  | |
| --- | --- |
| Database | Search strategy |
| PubMed | ((("peripheral"[All Fields] OR "peripherally"[All Fields] OR "peripherals"[All Fields] OR "periphereal"[All Fields] OR "peripheric"[All Fields] OR "peripherically"[All Fields]) AND ("insert"[All Fields] OR "insert s"[All Fields] OR "inserted"[All Fields] OR "inserter"[All Fields] OR "inserters"[All Fields] OR "inserting"[All Fields] OR "insertion s"[All Fields] OR "insertional"[All Fields] OR "insertions"[All Fields] OR "inserts"[All Fields] OR "mutagenesis, insertional"[MeSH Terms] OR ("mutagenesis"[All Fields] AND "insertional"[All Fields]) OR "insertional mutagenesis"[All Fields] OR "insertion"[All Fields]) AND ("central"[All Fields] OR "centrally"[All Fields] OR "centrals"[All Fields]) AND ("catheter s"[All Fields] OR "catheters"[MeSH Terms] OR "catheters"[All Fields] OR "catheter"[All Fields])) OR "PICCs"[All Fields]) AND ("central venous catheters"[MeSH Terms] OR ("central"[All Fields] AND "venous"[All Fields] AND "catheters"[All Fields]) OR "central venous catheters"[All Fields] OR ("central"[All Fields] AND "venous"[All Fields] AND "catheter"[All Fields]) OR "central venous catheter"[All Fields] OR "CVCs"[All Fields]) AND ("parenteral nutrition, home"[MeSH Terms] OR ("parenteral"[All Fields] AND "nutrition"[All Fields] AND "home"[All Fields]) OR "home parenteral nutrition"[All Fields] OR ("home"[All Fields] AND "parenteral"[All Fields] AND "nutrition"[All Fields]))  Identified 58 articles, Date: June 3. 2025. |
| Embase | ('peripherally inserted central catheter'/exp OR 'peripherally inserted central catheter' OR (peripherally AND inserted AND ('central'/exp OR central) AND ('catheter'/exp OR catheter)) OR piccs) AND ('central venous catheter'/exp OR 'central venous catheter' OR (('central'/exp OR central) AND venous AND ('catheter'/exp OR catheter)) OR cvcs) AND ('home parenteral nutrition'/exp OR 'home parenteral nutrition' OR (('home'/exp OR home) AND parenteral AND ('nutrition'/exp OR nutrition)))  Identified 262 articles, Date: June 3. 2025. |
| Cochrane Library | ((peripherally inserted central catheter): ti,ab,kw OR (PICCs):ti,ab,kw OR MeSH descriptor: [peripherally inserted central catheter] in all MeSH products) AND ((central venous catheter):ti,ab,kw OR (CVCs):ti,ab,kw) OR MeSH descriptor: [central venous catheter] explode all trees)AND ((home parenteral nutrition):ti,ab,kw OR MeSH descriptor: [home parenteral nutrition ] explode all trees)  Identified 3 articles, Date: June 3. 2025. |
