# Supplementary material for: Comparison of catheter-related bloodstream infection between peripherally inserted central catheters and tunneled central venous catheters in patients receiving home parenteral nutrition: a meta-analysis
Source: Front Nutr. 2026 Feb 17;13:1742418. doi: 10.3389/fnut.2026.1742418 (PMC12953089; doi:10.3389/fnut.2026.1742418)
Supplement: Supplementary file 2 [file Table_2.DOCX]

**Supplementary Table S2. Quality assessments of studies based on Newcastle Ottawa Scale.**

| **First author** | **Is the case definition adequate?** | **Representativeness of the cases** | **Selection of controls** | **Definition of controls.** | **Control for important factors** | **Exposure assessment** | **Same method of ascertainment for cases and controls.** | **Non-response rate** | **NOS score** |
| --- | --- | --- | --- | --- | --- | --- | --- | --- | --- |
| Vashi | 1 | 0 | 0 | 1 | 1 | 1 | 1 | 1 | 6 |
| Santacruz | 1 | 0 | 0 | 1 | 1 | 1 | 1 | 1 | 6 |
| Botella-Carretero | 1 | 0 | 0 | 1 | 1 | 1 | 1 | 1 | 6 |
| Elfassy | 1 | 0 | 0 | 1 | 1 | 1 | 1 | 1 | 6 |
| Touré | 1 | 0 | 1 | 1 | 1 | 1 | 1 | 1 | 7 |
| Durkin | 1 | 0 | 0 | 1 | 1 | 1 | 1 | 1 | 6 |
| Cotogni | 1 | 0 | 1 | 1 | 1 | 1 | 1 | 1 | 7 |
| Christensen | 1 | 0 | 0 | 1 | 1 | 1 | 1 | 1 | 6 |
| Xue | 1 | 0 | 0 | 1 | 1 | 1 | 1 | 1 | 6 |
| Konrad | 1 | 0 | 0 | 1 | 1 | 1 | 1 | 1 | 6 |
