# Supplementary material for: Comparison of catheter-related bloodstream infection between peripherally inserted central catheters and tunneled central venous catheters in patients receiving home parenteral nutrition: a meta-analysis
Source: Front Nutr. 2026 Feb 17;13:1742418. doi: 10.3389/fnut.2026.1742418 (PMC12953089; doi:10.3389/fnut.2026.1742418)
Supplement: Supplementary file 3 [file Table_3.DOCX]

**Supplementary Table S3 GRADE score.**

| **Quality assessment** | | | | | | | **Summary of Findings** | | | | |
| --- | --- | --- | --- | --- | --- | --- | --- | --- | --- | --- | --- |
| **Participants (studies) Follow up** | **Risk of bias** | **Inconsistency** | **Indirectness** | **Imprecision** | **Publication bias** | **Overall quality of evidence** | **Study event rates (%)** | | **Relative effect** (95% CI) | **Anticipated absolute effects** | |
|  |  |  |  |  |  |  | **With Control** | **With CRBSI** |  | **Risk with Control** | **Risk difference with CRBSI** (95% CI) |
| **CRBSI** (CRITICAL OUTCOME) | | | | | | | | | | | |
| 1885 (10 studies) | serious^1^ | serious^2^ | very serious^3^ | serious^4^ | undetected | ⊕⊝⊝⊝ **VERY LOW**^1,2,3,4^ due to risk of bias, inconsistency, indirectness, imprecision | 208/615  (33.8%) | 131/1270  (10.3%) | **OR 0.2**  (0.13 to 0.31) | **Study population** | |
|  |  |  |  |  |  |  |  |  |  | **338 per 1000** | **245 fewer per 1000** (from 201 fewer to 276 fewer) |
|  |  |  |  |  |  |  |  |  |  | **Moderate** | |
|  |  |  |  |  |  |  |  |  |  | **228 per 1000** | **172 fewer per 1000** (from 144 fewer to 191 fewer) |

^1^ Small sample size, observational study
^2^ I2 heterogeneity is relatively large.
^3^ Inconsistent evaluation criteria for outcome indicators; inclusion of various study populations
^4^ The incidence rate of the event is relatively low.
